# Response to “Introduction of the EMR-integrated I-PASS ICU Handoff Tool”

**DOI:** 10.1097/pq9.0000000000000333

**Published:** 2020-07-23

**Authors:** Lauren M. Tufts

**Affiliations:** From the Marshall Pediatrics, Huntington, WV.

Although the transition of patient care is performed routinely within the healthcare system, such transitions pose a great risk of sentinel events and the associated patient morbidity.^1^ Therefore, the achievement of quality patient handoff through avoidance of miscommunication is essential in improving patient care. Communication errors during handoff can be significantly decreased by utilizing a structured handoff tool or mnemonic, such as I-PASS.^2^ Our study *Addition of CORES to the I-PASS Handoff: A Resident-led Quality Improvement Study* describes the integration of CORES (Seattle, Wash.), a third-party program that formulates patient lists centered around the I-PASS mnemonic, into Cerner (North Kansas City, Mo.), our organization’s electronic medical record (EMR) system.^3^ Our team integrated and implemented CORES to improve resident-to-resident handoff quality and, in turn, improving patient care.

As described, there is a high risk of miscommunication during handoff between members of the same care team.^1^ Similarly, the transition of care between separate care teams, such as in OR to pediatric intensive care unit (PICU) patient transfers, poses a high risk of patient morbidity as a direct result of the handoff error.^4^ Studies show that the use of a structured, direct OR to PICU transfer process leads to a reduction in the handoff communication error rate, fewer handoff information omissions, and improvement in handoff efficiency.^5^

With this knowledge, it is with great interest and excitement that we discover Caruso et al.’s^6^ utilization of I-PASS during OR to PICU transfers. Their work speaks to the teams’ ability to recognize a great source of potential patient morbidity, miscommunication, and inefficiency, and find a solution for such problems. Caruso et al. did so by engineering a novel handoff tool modeled from I-PASS and integrating this tool into their EMR, EPIC (Verona, Wis.), to be used during OR to PICU transfers. By creating this internally developed I-PASS handoff tool, Caruso et al. were able to find benefit through the utilization of structured handoff systems while mitigating the risks associated with third-party applications, such as CORES. For this, we applaud and respect Dr. Caruso and his team. The innovation of novel tools such as this is indeed imperative for the development of safer handoff and subsequent improvement in patient care.

## DISCLOSURE

The author has no financial interest to declare in relation to the content of this article.
